# Multiple anaplastic lymphoma kinase-positive primary inflammatory myofibroblastic tumors with spontaneously expanding and shrinking nodules in both lungs: a case report

**DOI:** 10.1186/s44215-024-00153-7

**Published:** 2024-05-21

**Authors:** Shunsuke Nomura, Mitsuhito Kaji, Nobuyuki Shiina, Ryohei Chiba, Yasushi Cho, Haruhiko Shiiya, Tatsuya Kato

**Affiliations:** 1 Department of Thoracic Surgery, Sapporo Minami-Sanjo Hospital, 4-2 S3W6, Chuou-Ku, Sapporo, Hokkaido 060-0063 Japan; 2 Department of Thoracic Surgery, Sapporo-Kosei General Hospital, N3E8, Chuou-Ku, Sapporo, Hokkaido 060-0033 Japan; 3 Department of Thoracic Surgery, Hokkaido University Hospital, N14W5, Kita-Ku, Sapporo, Hokkaido 060-8648 Japan

**Keywords:** Inflammatory myofibroblastic tumor, Multiple, Anaplastic lymphoma kinase

## Abstract

**Background:**

Inflammatory myofibroblastic tumors (IMTs) are uncommon neoplasms most prevalent in individuals under 40 years old and predominantly in the lungs. Despite their rarity, multiple anaplastic lymphoma kinase (ALK)-positive IMTs, especially those of various sizes, have not been widely reported. This report describes a case of multiple ALK-positive IMTs in the lungs, aiming to further our understanding of their behavior and management.

**Case presentation:**

Herein, we present the case of a 64-year-old woman who presented with an abnormal shadow on chest examination. Chest computed tomography revealed a main tumor in the right middle lobe and multiple irregularly shaped small nodules in both lungs. Thus, thoracoscopic wedge resection of the left lower lobe was performed for diagnosis. Pathological findings indicated smooth muscle proliferation without malignancy. IMT was diagnosed following thoracoscopic right middle lobectomy. Twenty months postoperatively, one residual nodule shrank, but another grew.

**Conclusions:**

This is the first report of multiple ALK-positive IMTs in both lungs, highlighting the need for definitive diagnosis and treatment of IMTs based on surgical resection. Although caution is required in patients with lymph node metastases or distant metastases, careful follow-up is acceptable unless there is a tendency for nodules to increase in size on imaging.

## Background

Inflammatory myofibroblastic tumors (IMTs) are rare neoplastic lesions characterized by spindle cell proliferation, the presence of myofibroblasts, and marked infiltration of inflammatory cells, such as lymphocytes and plasma cells. The incidence of IMT ranges from 0.04 to 0.3% of lung tumors [1, 2], and they are most commonly found in patients under 40 years old [1]. The lung is the most common primary site, followed by intraperitoneal development [3], which varies widely throughout the body. Anaplastic lymphoma kinase (ALK) gene rearrangements on chromosome 2p23 have been observed in 56% of IMT cases [4]. In addition, IMT often presents with isolated nodules on imaging, with relatively few reports of multiple nodules (5–11%) [2, 5]. Herein, we describe a case of multiple ALK-positive IMTs in both lungs that increased and decreased in size over time.

## Case presentation

**Fig. 1:**
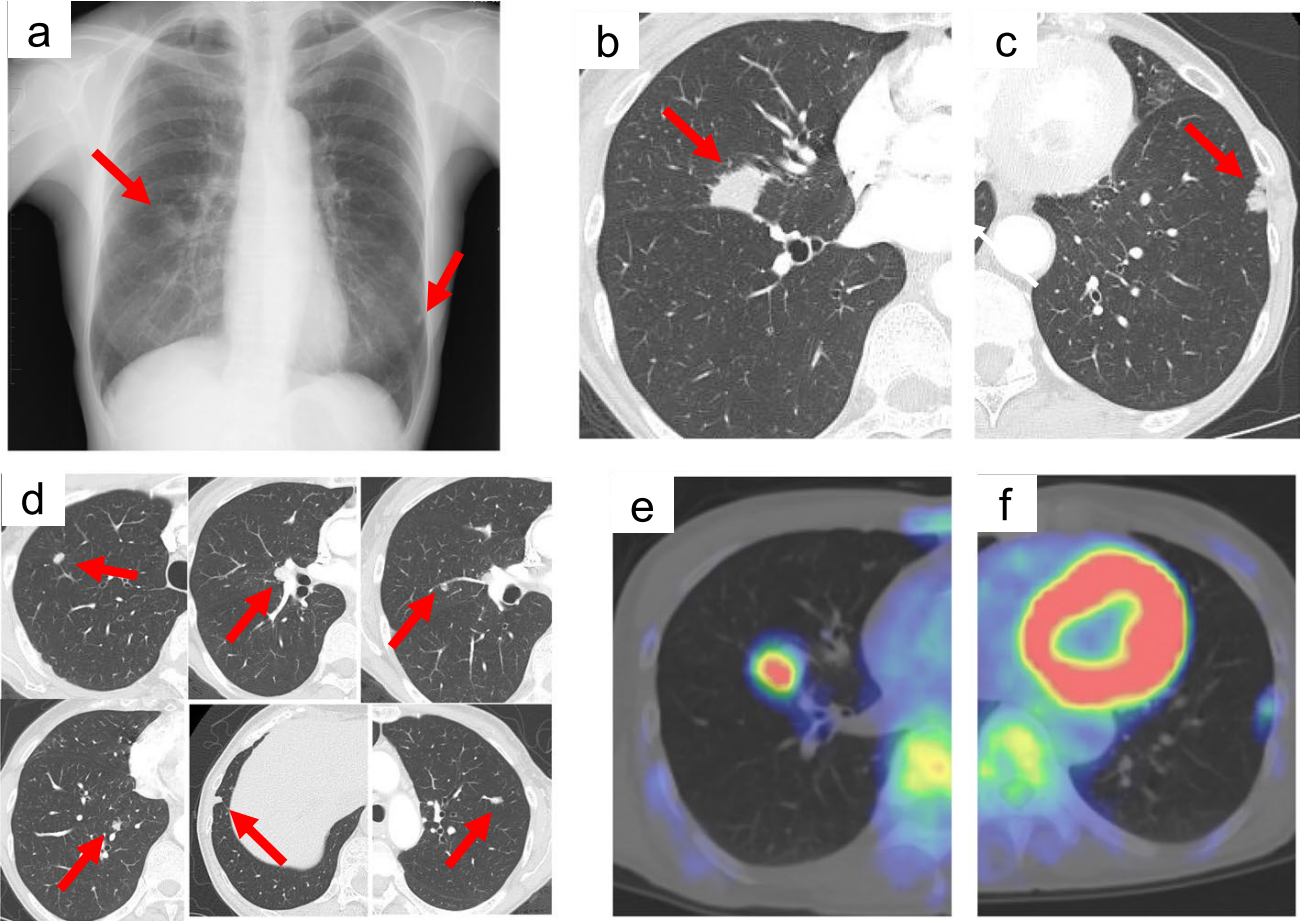
**a** Chest X-ray findings showing nodular shadows on the right middle and left lower lung field (arrows). **b** Chest CT showing a 1.8 × 1.6 cm nodule in the right middle lobe (arrow). **c** A 1.0 × 0.7 cm nodule in the left lower lobe (arrow). **d** Small nodules in both lungs (arrows). **e** FDG-PET-CT findings showing an increase in FDG accumulation in the nodule located in the right middle lobe (SUV_max_: 2.9). **f** An increase in FDG accumulation in the nodule located in the left lower lobe (SUV_max_: 1.1). CT, computed tomography; FDG, fluorodeoxyglucose; PET, positron emission tomography

A 64-year-old woman presenting with no chief complaint exhibited an abnormal shadow on chest examination. The patient had a history of smoking 20 cigarettes/day for 10 years. Chest computed tomography (CT) revealed multiple irregular nodules in both lungs; therefore, the patient was referred to our hospital for further examination. On admission, the patient’s height and weight were 152.7 cm and 38.2 kg, respectively, and there were no abnormalities on chest auscultation. No superficial lymph nodes were palpable, and there were no abnormalities in blood chemistry or blood count, including carcinoembryonic antigen, cytokeratin 19 fragment, squamous cell carcinoma antigen, sialyl Lewis X antigen, and pro-gastrin releasing peptide. Plain chest radiography revealed an irregular nodule of 1.8 cm in size in the right middle lobe and an irregular nodule of 1.0 cm in size in the left lower lobe (Fig. 1a). Chest CT revealed an irregular nodule with an irregular concentration of 1.8 × 1.6 cm in diameter adjacent to the interlobar visceral pleura in the lateral segment of the right middle lobe (Fig. 1b); an irregular nodule with an irregular margin of 1.0 × 0.7 cm in diameter adjacent to the pleura in the anteromedial basal segment of the left lower lobe (Fig. 1c); and other small nodules in both lungs (Fig. 1d). Enlargement of the mediastinal and hilar lymph nodes was not observed. Positron emission tomography (PET)-CT revealed an accumulation of fluorodeoxyglucose (FDG) with a maximum standard uptake value (SUV_max_) of 2.9, consistent with the right middle lobe nodule, and another with an SUV_max_ of 1.1, consistent with the left anteromedial basal segment nodule (Fig. 1e, f). Bronchoscopy revealed abrasion cytology class I from the right middle bronchus. For diagnostic purposes, surgery was performed on the left lower lobe nodule.

**Fig. 2:**
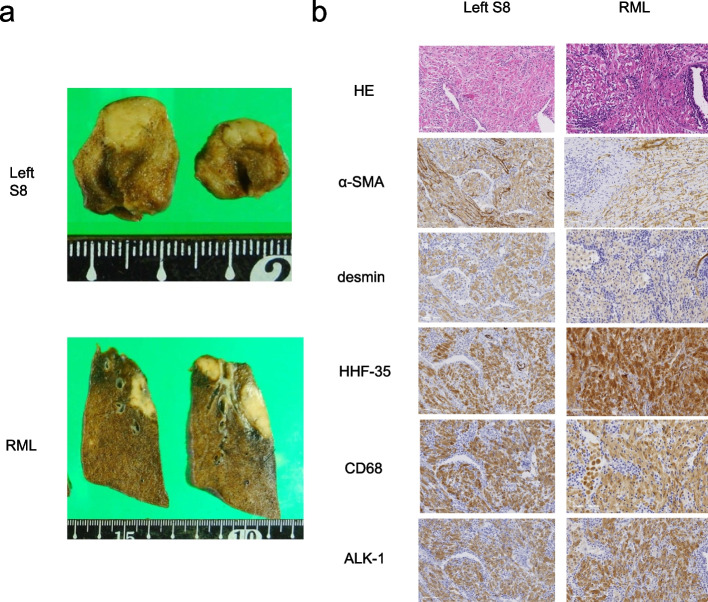
**a** The resected specimen of the anteromedial basal segment (S8) of the left lower lobe showing a grayish-white solid nodule measuring 1.0 cm in diameter. The resected specimen of the right middle lobe showing three yellowish-white solid nodules measuring 2.5 cm, 1.0 cm, and 0.4 cm in diameter, respectively. **b** Microscopic images stained with hematoxylin and eosin showing spindle-shaped cells of the cytoplasm acidophil (× 20). Both nodules were positive for α-SMA, desmin, HHF-35, CD-68, and ALK-1 (× 20). RML, right middle lobe; α-SMA, α-smooth muscle actin; HHF-35, muscle actin; ALK-1, anaplastic lymphoma kinase-1

**Fig. 3:**
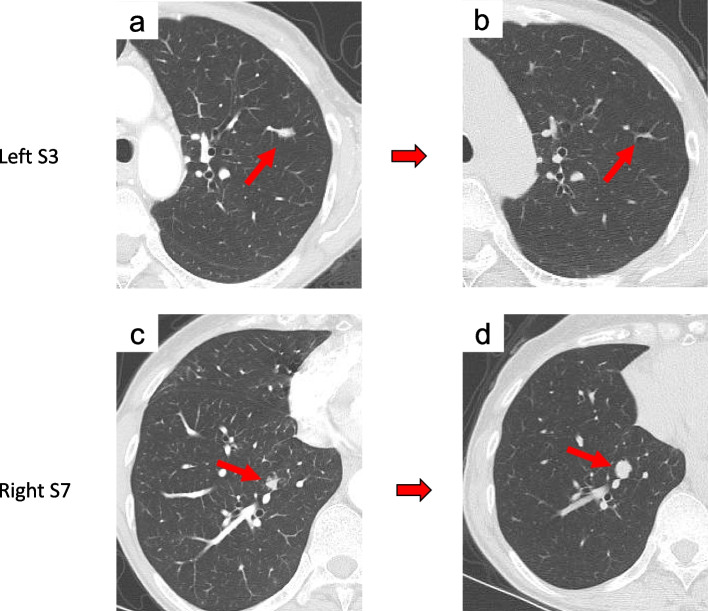
**a**, **b** Twenty months after surgery, the nodule (arrow) in the anterior segment of the left upper lobe had shrunk. **c**, **d** The nodule (arrow) in the medial basal segment of the right lower lobe had increased in size. S3, the anterior segment of the upper lobe; S7, the medial basal segment of the right lower lobe

Video-assisted thoracoscopic wedge resection of the left lower lobe was performed under general anesthesia. The resected specimen contained a light brown solid nodule measuring 1.0 × 0.7 × 0.5 cm (Fig. 2a). Histopathological examination revealed eosinophilic spindle-shaped cells in the lung parenchyma beneath the visceral pleura. The lesion contained a sporadic luminal structure composed of the alveolar epithelium and infiltrated plasma cells. Spindle cells were immunostained with α-smooth muscle actin (α-SMA) and were weakly positive for desmin and partially positive for human melanoma black-45 (Fig. 2b). Therefore, smooth muscle proliferation was diagnosed. Since there were no malignant findings, a right middle lobectomy was performed. The rapid pathologic diagnosis was inflammatory lesions, and lymph node dissections were not performed. The resected specimen exhibited solid yellowish-white nodules of 2.5 × 2.4 × 1.2 cm, 1.0 × 0.7 cm, and 0.4 × 0.3 cm (Fig. 2a). Histopathological examination revealed eosinophilic proliferation of short spindle-shaped cells, lymphoid infiltration, and lymphoid follicular formation. The spindle-shaped cells were immunostained for vimentin, CD68, muscle actin (HHF-35), and ALK-1. Weakly positive α-SMA and desmin staining was also observed (Fig. 2b). Based on these findings, the patient was diagnosed with primary pulmonary IMT. Subsequently, immunostaining of the left tumor revealed HHF-35, CD68, and ALK-1 positivity, and this tumor was also diagnosed as IMT (Fig. 2b). The patient’s postoperative course was uneventful. Twenty months postoperatively, the nodule in the left upper lobe had shrunk, while the nodule in the right lower lobe had grown, although the other nodules did not change in size (Fig. 3a–d). Currently, the patient is under outpatient observation without treatment.

## Discussion

To our knowledge, this is the first reported case of multiple ALK-positive IMTs with spontaneously shrinking and expanding nodules in both lungs. Previously, lesions diagnosed as inflammatory pseudotumors were classified as organizing pneumonia, fibrous histiocytoma, or lymphoplasmacytic type [6]. Tumors exhibiting proliferation of spindle cells characterized by myofibroblasts and marked lymphocytic and plasma cell infiltration were defined as IMT [7], which often follows a benign clinical course. However, there have been reports of ALK gene rearrangements, and ALK gene expression has been associated with local recurrence [4], suggesting that IMT is not the result of an inflammatory response but rather a tumor lesion. Images often show well-defined solitary nodules, scarce calcifications, and cavitation [3]. PET shows FDG accumulation that can make it difficult to differentiate IMT from other malignancies [8]. Inflammatory cells, such as lymphocytes and macrophages, take up large amounts of FDG, and fibroblasts also ingest high levels of glucose at the time of proliferation, suggesting that IMT is associated with variable SUVs, depending on the degree of inflammation in the tumor. To our knowledge, only two case reports have discussed IMT occurring exclusively in the lungs, and both patients were negative for ALK [9, 10].

In our case, distinguishing between primary IMT lesions and metastatic nodules was challenging due to the inherent limitations of definitive evidence. Our classification of the nodules as primary IMTs rather than metastases was based on a comprehensive assessment of clinical and radiological findings, not on definitive pathological or molecular markers. The absence of extrapulmonary metastatic lesions and the similar histopathological characteristics among the nodules support this classification. Nonetheless, without specific genetic or molecular markers to conclusively identify their origins, this distinction is a clinical judgment based on the available evidence. This highlights the complex nature of diagnosing IMTs and the importance of cautious interpretation of changes in nodule size, including reduction, which alone does not definitively indicate IMT. Histopathological and immunohistochemical findings supported the initial diagnoses, but the absence of re-biopsy or further molecular analysis of the smaller nodule means we cannot definitively classify it as IMT. This underscores the need for ongoing research into the molecular and genetic aspects of IMTs to establish more precise criteria for distinguishing between primary and metastatic lesions.

A histopathological examination revealed inflammatory cell infiltration, myofibroblast-derived spindle cells, lymphocytes, plasma cells, and collagen fibrosis of the stroma. Immunohistological examination revealed an absence of an epithelial marker, high levels of the mesenchymal markers vimentin and α-SMA, and positive results for desmin, HHF35, and cytokeratin [7]. Because IMTs are predominantly infiltrated by various inflammatory cells, they are unlikely to be diagnosed preoperatively. In addition, the accuracy rate of intraoperative frozen diagnosis is unlikely to be high. In this case, the right middle lobe nodule was diagnosed as an inflammatory lesion without a malignant finding during rapid intraoperative diagnosis. Resection was also considered necessary for a definitive diagnosis, and immunostaining for ALK aided in the diagnosis. Differentiation of multiple tumors may also be useful, as presented in the current case. While the occurrence of multiple ALK-positive IMT nodules is rare and not typically concerning due to ALK-positive IMT’s invasive nature, our case underlines the diagnostic challenges of such cases.

Complete surgical resection is the cornerstone of IMT treatment. When complete resection is possible, wedge resection has been regarded as sufficient [2]. However, because local recurrence and metastases have been reported, it is necessary to obtain a sufficient margin of resection. Previous studies have reported 5-year and 10-year survival rates of 91.3% and 77.7%, respectively, when complete resection has been achieved [1]. However, the type of surgery varies, and a sufficient number of patients must be treated to more accurately predict prognosis. Other therapies include radiotherapy [1], chemotherapy [11], steroid therapy [10], and nonsteroidal anti-inflammatory drugs alone [12]. However, there is no established therapy for lung IMT. Many patients exhibit rearrangements of the ALK gene, which codes for one of the tyrosine kinase receptor proteins. Thus, the tyrosine kinase inhibitor, crizotinib, has also been considered effective in treating IMT [13]. However, caution should be exercised in some cases, including those involving lymph node metastases [14], distant metastases, and sarcomatous transformation [15]. In our patient’s case of incomplete IMT resection, treatment decisions, including the consideration of ALK inhibitors like crizotinib, were complex due to the nodules’ ALK positivity and the patient’s stable state. The benign and non-aggressive nature of the nodules led to a conservative initial approach, highlighting the need for a multidisciplinary strategy that balances surgical, medical, and observational methods with the patient’s condition and treatment risks.

In conclusion, the IMT diagnosis was confirmed through pathology and ALK positivity, marking this as the first case of multiple ALK-positive IMTs in both lungs. Ongoing careful monitoring is essential.

## Data Availability

Data sharing is not applicable to this article, as no datasets were generated or analyzed during the current study.

## Abbreviations

IMT: Inflammatory myofibroblastic tumor
ALK: Anaplastic lymphoma kinase
CT: Computed tomography
PET: Positron emission tomography
FDG: Fluorodeoxyglucose
SUV: Standard uptake value
SUV_max_: Maximum SUV
α-SMA: α-Smooth muscle actin
